# International Dental Federation and International Commission of Education: Second General Meeting, Held at Stockholm, Sweden, 1902

**Published:** 1903-01

**Authors:** 


### INTERNATIONAL DENTAL FEDERATION AND INTERNATIONAL COMMISSION OF EDUCATION: SECOND GENERAL MEETING, HELD AT STOCKHOLM, SWEDEN, 1902.

(Continued from Vol. XXIII., page 853.)

### INTERNATIONAL DENTAL FEDERATION.

*Wednesday, August 20, 1902.*

Dr. E. Sauvez, the secretary-general, then read the report which here follows:

Gentlemen and Honored Confrères,—I consider that my first duty before presenting this report is to express my deep gratitude to my colleagues of the Executive Council for the honor conferred upon me last year at the London and Cambridge meetings by appointing me secretary-general of the International Dental Federation for the year 1901-2. I fully appreciate the importance of the position, and if during the course of the past year I have given evidence of even slight negligence, I now ask you to excuse anything that I may have done that could deserve such a criticism. Several members of the Commission of Education because of insurmountable circumstances are not able to be present at these meetings, and I have been requested to present the regrets of Drs. Kirk, Roy, Pearson, Rosenthal, and Burne.

I will now give you a *résumé* of the most important events that brought about the organization of this body, and also the steps taken by the Federation since its last year's meeting. It is exactly two years since the Federation was organized, it being created by a resolution of the general assembly of the Third International Dental Congress, held in Paris in 1900. Its first meeting took place last year in Cambridge, and in looking at the present audience I cannot help seeing that the importance of this body has been largely increased since our last meeting. The great assembly of which the Federation is the outgrowth thought that the work of preparing congresses by the constitution of international committees should survive for a future work similar in nature to that for which they were originally created. That assembly appreciated the advantages to be derived from bringing together the professional forces scattered in the different countries and forming in this way a permanent international body. This is a very solid and compact group because of the status of the members that compose it, inasmuch as the members of the national committees have been elected by their *confrères* after due selection, the only way of satisfying minds imbued with modern liberal ideas. For this reason the Federation met with warm approval beyond the Atlantic, as it is an organization fully in harmony with the ideas of the sons of free America.

The Federation, then, and its Executive Council are the direct outgrowth of the Congress of 1900. At the memorable meeting to which we have already referred the Fédération Dentaire Internationale was created, and it was decided that a permanent committee of nine members should direct the work of the Federation until the holding of the next congress. The Executive Council decided to create international commissions to study the most important questions of our profession,—viz., dental education and public dental hygiene. The first session was held last year, partly in London and partly in Cambridge. That session was devoted especially to the organization of the work and to the consideration of all the questions to be submitted for your study and consideration, and to the appointment of essayists to discuss these questions.

The foregoing is a *résumé* of the events that took place up to the adjournment of the Cambridge meeting. Let us examine now what has occurred since that time. The secretary has published the proceedings of the London-Cambridge meeting,—viz., a *résumé* of the meetings of the Executive Council and of the International Commission of Education. This report has been published in French, and, thanks to the kind interest which Dr. Kirk had in this matter, the report has also been published in English, and each of you have received a copy. We were also able to publish the report in German, and our colleague, Dr. Aguilar, has taken charge of the publication of the report in Spanish. During the course of the last year we have at intervals published bulletins in four different languages so that the dental profession might be made acquainted with the most important features of our work. Lastly, we have carried out several decisions of the Executive Council, and we have had the satisfaction of receiving a considerable number of replies. Dr. Roy, secretary of the Commission of Education, in conjunction with the secretary-general, has taken steps to obtain the reports the collection of which was decided upon at the last meeting. The Executive Council had asked that a report of the professional status of each country with special reference to schools, societies, and journals be forwarded to the secretary-general. The Commission of Education asked that the members appointed for this purpose should answer the three questions that had been submitted with reference to the best methods of education. This Commission also requested that reports be prepared on the subject of dental legislation and education, and to state the conditions under which the affiliation of all the schools to the Commission of Education of the Fédération Dentaire Internationale might be brought about. The members of the Commission on Public Dental Hygiene were also requested to present a report upon dental hygiene with reference to public health. We have received, translated, and published in three fasciculi which have been sent to you the communications which were forwarded to us, and we now take the opportunity to thank the essayists for their careful work.

We are in receipt of a communication in which we are informed that Dr. Franck had been regularly appointed by the general assembly comprising the delegates of several dental societies of Austria and Hungary in place of Dr. Pichler, consequently Dr. Franck is now a member of the Executive Council, and his collaboration is of great importance to the work of the Federation.

The officers of the Executive Council have held several meetings at frequent intervals and numerous decisions have been rendered. The question of deciding upon the date of this meeting was the cause of numerous discussions and of a voluminous correspondence. The officers of the Federation and of the American Dental Society of Europe, wishing to hold a joint meeting, met with considerable difficulty in fixing the date because of the meetings of the French Congress at Montauban and the German Congress in Munich. Finally, through the efforts of several officers of both societies and the personal efforts of Messrs. Royce, Mitchell, Cunningham, and Davenport, we were able to agree, as the only purpose in view was to assure the success of the Stockholm meeting and a perfect understanding between the members who have come from such distant countries. Because of these circumstances we were obliged to decline an invitation which had been addressed to the members of the Federation by Dr. Haderup, of Copenhagen, on behalf of the Danish Dental Society.

The Executive Council has done everything within its power to increase the attendance at the Stockholm meeting. Communications were addressed to Dr. Limberg, president of the Russian National Committee, requesting that the Russian societies should send delegates to the Commission of Education. This question was discussed at the Congress of Odessa, and we have been informed that Dr. Klingelhöfer, of St. Petersburg, has been appointed delegate. Professor Limberg, because of ill health, will not be able to be with us on this occasion. We are glad to say that we have been informed that from fifty to sixty delegates have arrived, a number considerably larger than that of last year.

This completes the description of the most important features of our work during 1901-2.

Enough has been said with reference to the past, therefore we will now survey the present. The International Dental Federation begins to-day its second meeting in this beautiful city of Stockholm, in accordance with the decision passed last year at Cambridge and accepted unanimously by the members of the Executive Council, following the invitation regularly transmitted to us by Messrs. Förberg, Christensen, Sandstedt, and Forssman.

The Executive Council will consider whether there is any necessity for amending the by-laws, inasmuch as it is only one year since they were adopted. It will look into the question of the proportional division of the expense incurred by the Federation. It will have to appoint officers for the coming year, and it will have to carefully examine the question of the part which the Federation should take in the next International Congress of Medicine, to be held in Madrid in April, 1903. The Federation will have to designate the city in which the next meeting of our association should be held, and as the period for the holding of the next International Congress is very near, it will have to select the city in which this international gathering shall take place. It will have to appoint to the existing committees the delegates to the Federation. The Executive Council cannot make this distribution of members, which by the way is the basis of the work of this association, unless the Executive Council knows definitely the names of the delegates, of the societies they represent, as well as the character of the mission they have been intrusted to carry out in Stockholm. In one word, the Executive Council should know the special line of work the delegates would wish to take up in connection with the Federation work.

We have two official delegates from foreign governments. Dr. Florestan Aguilar is the representative of Spain, and Dr. Vincenzo Guerini represents the government of Italy. We have received official communications from several societies with reference to the appointment of delegates, but some of the delegates that are in Stockholm at present have not registered as yet, and it is necessary that the Executive Council should certify their credentials. I will therefore ask the delegates to turn over to the secretary-general their credentials immediately after the adjournment of this meeting; also the papers, addresses, etc., with which they have been intrusted by their governments, federations, or societies. They will be asked to indicate in what committee they would like to take part. All of you, I presume, understand the importance of this recommendation.

It will also be the duty of the Executive Council to discuss whether it would be useful to appoint new committees in order to widen the field of activty of the Federation. We believe that it would be a useful measure to create a committee on schools that would regulate all the questions regarding scholarship, matriculation, requirements of admission, and examination of students. Such a committee would be in position to give correct information regarding all topics connected with school organization. It would also be advisable to organize a committee on jurisprudence and deontology, in order to centralize in this way all the information regarding the practice of dentistry in the different countries, as well as all the laws and regulations that affect our profession. A committee on statistics would also be an advisable addition to the committees already appointed by the Federation.

These are, gentlemen and *confrères,* only very superficial indications, and it remains with the Executive Council to decide whether such committees should be appointed.

The work of the International Commission on Education will be carefully discussed in a very complete report which will be presented by Mr. Martinier, who was appointed on this Commission at the meeting held in August, 1900, and who had been substituted last year by Dr. Roy. In this report the essayist will examine the documents that are printed in the three fasciculi.

Twelve reports were received in time for publication in these bulletins; others arrived too late to be inserted therein. You will find in these reports profitable topics for discussion, and you will be able to reach definite conclusions with reference to the best methods of dental education. This Commission, before proceeding with its work, will have to appoint officers for the coming session.

The Commission on Public Dental Hygiene submitted a series of questions to representative members of the dental profession the world over, as appears in the report which has been translated and published in French. Unfortunately we have not had time to translate and publish all the papers and documents connected with this report. The commission will increase its membership by the addition of new delegates, will appoint officers, and will organize its general plan of work. We are convinced that the discussions of these two commissions will bring about fruitful results, and that the 1902 session of the Federation will do a great deal towards the solution of the multiple questions which will be brought up for discussion.

This, gentlemen, is the work that we will have to carry out in Stockholm. Besides that we will have to prepare the work for the next session, submit new topics for discussion, appoint essayists, and divide the work according to personal abilities and inclinations. It is in this way only that we will be able to organize a Federation which will grow daily in importance and usefulness.

I have attempted to bring before you in this report in as clear a manner as possible the work that is awaiting our consideration. I am sure that you all will agree with me when I say that the very few days that we will have for the study of these important points constitute very limited time if we consider the magnitude of the work.

In ending this report, I regret that it has been of such slight interest, but it is only a business report. Before concluding, however, I want to call your attention to another very important point in connection with our work,—viz., that nationalities and personalities do not exist here. As I stated last year, we are only the cells of an organ; the cells change, but the organ remains the same. The great army which is formed by all the dentists the world over has appointed us to represent it and to take care of their interests until the next meeting, that is, until the next congress. The members that are here present constitute the officers of this army, and we must show the next congress that we have endeavored to carry out this mission.

EXECUTIVE COUNCIL.

*First Extra Session, August 14, 1902.*

The meeting was called to order at 4 P.M. in the Grand Hotel at Stockholm, by the president of the Executive Council, Dr. Chas. Godon. The following members were present: Drs. Aguilar, Cunningham, Förberg, Harlan, Hesse, and Sauvez.

Dr. Sauvez made an *exposé* of the financial situation of the Federation, and stated that the expenses of the meetings at Paris, London, and Cambridge amounted to 1164.30 francs, and the receipts to 775 francs. He also stated that there were as yet a number of subscriptions to be collected, and that this would balance up the account. He then submitted to the several members of the Council his written report.

The Council then decided to appoint a committee to study the arrangements made last year with the publisher of the transactions of the Federation, and to prepare a contract for the publication of the transactions of the present session. Drs. Harlan and Frank were appointed as such committee.

Dr. Sauvez then stated that the expenses incurred in the preparation of the present session of the Federation amounted to 1295.65 francs, this sum including the cost of preparing and publishing the three fasciculi published by the Federation.

The Council appointed a committee composed of Drs. Aguilar and Sauvez to study the best way of defraying the expenses of the Federation meetings.

*Second Session, August 15.*

The meeting was called to order by President Godon at 10.30 A.M. The minutes of the last meeting were read and approved. Dr. Cunningham presented the regrets of Dr. J. E. Grevers, Amsterdam.

The committee on finance, appointed at the last meeting, presented a report. The committee believed that each member of the Council and of the different commissions should pay a fee of fifty francs, and that the delegates should pay a fee of twenty-five francs. They also advised the opening of a voluntary subscription among the several journals, federations, associations, and societies, and that the committees of organization of future congresses shall also contribute towards this fund. The committee also decided that when a member represents several societies, he should pay a separate fee for each such society. The Council adopted these propositions.

Dr. Florestan Aguilar was then unanimously elected treasurer of the International Dental Federation.

On motion by Dr. A. W. Harlan, Chicago, Dr. Truman W. Brophy was introduced, in order to present the several invitations relating to the Fourth International Dental Congress.

Drs. Förberg and Sauvez then made a few remarks regarding the opening session of the International Dental Federation to be held at the Caroline Institute.

The questions embodied in the invitations presented by Dr. Brophy were then discussed by the members of the Council.

*Third Session, August 16.*

At this afternoon session, the president called the meeting to order, and Dr. Sauvez, the secretary-general, read the minutes of the last session, which were approved.

The invitation of the National Dental Association of the United States to hold the Fourth International Dental Congress in St. Louis was again taken up for discussion. Dr. Cunningham proposed that the final decision should be taken at the next meeting of the Federation. Drs. Franck and Harlan asked that the final decision should be taken at the meeting of the Executive Council to be held August 18. The Council accepted this last proposition.

*Fourth Session, August 16.*

Dr. Chas. Godon, President of the Council, called the meeting to order at 11.30 A.M. The following members were present: Drs. Aguilar, Cunningham, Förberg, Franck, Harlan, Hesse, and Sauvez. The minutes of the last session were read and approved.

Dr. Sauvez read the list of the delegates of governments, federations, and societies. He also read a list of the members at that time in Stockholm. Dr. Sauvez read the names of those that had been proposed as members and adjunct members of the International Commission of Education and of the International Commission of Public Dental Hygiene.

After the Council had rectified these lists, the secretary-general read a letter relative to the appointment of Dr. Klingelhöfer as the representative of the government of Russia. Dr. Klingelhöfer was then appointed adjunct member of the Executive Council of the International Dental Federation.

*Fifth Session, August 16.*

Dr. Chas. Godon presided over the session, which was attended only by Drs. Aguilar, Franck, and Sauvez. After having approved the minutes of the previous session, the secretary-general proposed a programme for the meeting to be held August 18. This programme as submitted by Dr. Sauvez was approved by the Council, which then adjourned until August 18.

*Sixth Session, August 18.*

The meeting was called to order at twelve noon by the president, Dr. Godon. The meeting was attended by Drs. Aguilar, Cunningham, Förberg, Franck, Harlan, Hesse, and Sauvez. The minutes of the previous meeting were read and approved.

After a short discussion regarding the F. D. I. banquet, the question of the invitation of the National Dental Association of the United States to hold the Fourth International Congress in the city of St. Louis was brought up for discussion.

Dr. Cunningham moved that the reply to this invitation should be postponed until the next meeting of the F. D. I.

Dr. A. W. Harlan was willing that the decision should be postponed to the next session, in order to give time to examine a technicality in the invitation in question, but Drs. Hesse and Aguilar stated that it was preferable to take up the discussion at once, as the Council must take into consideration that the invitation had been addressed through regular channels and in due form.

Dr. Cunningham insisted that the final decision should be deferred until the next session of the International Dental Federation, thinking that perhaps by that time invitations from other countries might have been received.

The president then decided that independently of the question of accepting or declining the invitation, the Council should fix at that session the date of the next Congress. It was then submitted to the vote of the Council and was adopted, as all the votes except one were in favor of the president's suggestion.

Professor Hesse thought that the invitation to hold the next Congress in the city of St. Louis, in the United States of America, should not be accepted, and further, that his opinion would have

Dr. Cunningham's support. He regretted that his views would suggest an apparent lack of courtesy towards the American *confrères,* but he thought that the general interests of the International Dental Federation should be considered first, and that it was only with that purpose in view that he decided to recommend the non-acceptance of the invitation. He then stated that his specific reasons would be submitted to the Council in writing.

The president stated that this question should be very carefully studied and considered before reaching a decision.

Dr. Franck stated that after having conferred with his countrymen they had reached the conclusion to accept the cordial invitation made by the dental profession of America.

Dr. Sauvez expressed himself plainly and definitely as being in favor of accepting the invitation.

Dr. Förberg said that as the reasons given by the members who opposed the acceptance of the invitation were not in any way so important as those advanced by the members who were in favor of holding the Congress in St. Louis, the invitation should be accepted.

The president submitted the question to the individual vote of the members, with the result that the invitation was accepted by six votes against two. The Council then appointed a committee composed of Drs. Hesse, Aguilar, and Sauvez, to prepare the acceptance to the invitation addressed by the National Dental Association of the United States.

The Council then proceeded to the election of officers for the ensuing year. The result was as follows: President, Dr. Ch. Godon; vice-presidents, Drs. Aguilar and Franck. Dr. Sauvez was elected secretary-general by acclamation.

*Seventh Session, August 18.*

The president called the meeting to order and the following gentlemen answered the roll-call: Drs. Aguilar, Cunningham, Förberg, Franck, Hesse, and Sauvez. The minutes of the previous meeting were read and approved.

In accordance with Dr. A. W. Harlan's proposition, the Council decided that in the letter which would be addressed in reply to the invitation presented by Drs. Wm. C. Barrett, Truman W. Brophy, and Eugene H. Smith, in the name of the National Dental Association of the United States, it should be specified that Drs. Truman W. Brophy, William C. Barrett, Eugene H. Smith, W. Conrad, Gordon White, H. A. Smith, W. R. Windhorst, S. H. Guilford, and J. D. Patterson have been designated to form a committee to collaborate towards the organization of the Fourth International Dental Congress, as representatives of the Executive Council appointed by the Third International Dental Congress.

The secretary-general read a statement signed by Messrs. Hesse and Cunningham, that they could not give consent that the F. D. I. should lend its support to the Congress to be held in St. Louis in 1904, for the following reasons:

1. A lapse of four years after the last International Dental Congress is too short a period to warrant the holding of another congress, as the progress which has been made in dentistry since then is not considerable enough to justify the holding of another international gathering.

2. That they could not assure the attendance of a sufficient number of members from each country to justify calling it an International Congress.

The secretary-general then read the letter to be addressed to the National Dental Association of the United States in reply to the invitation to hold the next congress in the city of St. Louis.

The document referred to is as follows:

The Executive Council of the International Dental Federation, intrusted by the Third International Dental Congress with the organization of the succeeding congress, having read the invitations adressed by—

The National Dental Association of the United States of America,
The governor of the State of Missouri,
The mayor of the city of St. Louis,
The National Association of Dental Examiners,
The Dental Society of the State of Missouri,
The St. Louis Dental Society,
The Society of Dental Science of St. Louis,
The Odontographs of Western Missouri and Eastern Kansas,
The St. Joseph Dental Society, these being regularly represented at the Stockholm sessions by Messrs. Truman W. Brophy, Wm. C. Barrett, and Eugene A. Smith, accredited for this purpose:

Considering, on the one hand,—

1. That the progress of dentistry in the interval of four years seems insufficient to warrant a scientific interest in a fourth International Dental Congress after the one that was held in Paris in 1900;

2. That it would have been desirable that the forthcoming congress should be held in a country in which a similar international reunion had not been held before:

But, on the other hand,—

3. That universal expositions attract a considerable number of visitors from all countries, and are especially favorable to the success of international congresses;

4. That several international congresses will be held upon the occasion of the St. Louis Universal Exposition, and that it is necessary that odontology should be represented;

5. That no other invitation has been received;

6. That it is desirable to reach a decision so as to give ample time for a good preparation;

7. That the good organization of the congress is assured by the worthiness of the invitations received:

ACCEPTS the invitations, and DECIDES that the Fourth International Dental Congress shall be held in the month of August, 1904, in the city of St. Louis, Missouri, United States of America.

The undersigned certifies that this is an extract of the proceedings of the Executive Council, and that it is an exact copy of the letter to be sent in reply to the invitation received.

The Secretary-General,
(Signed) DR. SAUVEZ.

The secretary-general, Dr. Sauvez, then read a letter from Dr. A. F. Caro, minister of Spain at Stockholm, requesting the Federation to take part in the Fourteenth International Congress of Medicine to be held in Madrid. The Executive Council addressed a letter of thanks to Minister Caro, assuring him that the Section on Odontology of the International Congress of Medicine to be held at Madrid would have the support of the F. D. I. The Council recommended the foreign representatives to take part in this section of the next International Congress of Medicine.

The question of deciding upon the city in which the next meeting of the F. D. I. should be held was then taken up for discussion.

Dr. Aguilar suggested that the Federation should hold its forthcoming meeting in the city of Madrid at the time of the holding of the Fourteenth International Congress of Medicine. A motion to this end was presented by Dr. Aguilar, and was adopted by seven votes against one.

Dr. A. W. Harlan made a motion to the effect that in case of death of one of the members of the Congress Committee, the other members should have the power to fill the vacancy. This motion was adopted.

*Eighth Session, August 19.*

The session was called to order by President Godon, the following members being present: Drs. Aguilar, Cunningham, Förberg, Franck, Hesse, and Sauvez. The minutes of the last session were read and approved.

The secretary-general proposed the names of Drs. Frick, Guye, and Roy for adjunct members in the International Commission of Education, the name of Dr. Heidé as a regular member of the Commission of Public Dental Hygiene, and Dr. Bödecker for adjunct member of the same commission. This proposition was adopted by the Council.

The committee on publication then presented its report relative to the publication of the transactions of the F. D. I.

Dr. Aguilar, treasurer of the F. D. I., presented a report in which it was shown that the Federation had a balance of 402.65 francs to its credit. The treasurer's report was approved.

The secretary-general then read the following communication from Dr. Haderup: "Dr. Haderup is of the opinion that a committee of three should be appointed, said committee to have power to increase its number, for the purpose of preparing an international dental nomenclature and stenography. This committee to present a report at the next meeting of the International Dental Federation, to be held in 1904."

The secretary-general also read a communication from Dr. Förberg, with reference to the publication in each country of periodical reports of the most important writings on dentistry recently published. This report could be submitted to an editorial committee which would publish them in a universal dental review in two or more languages. The bulletin of the Federation could be used for this purpose, and in that way the International Dental Federation which unites to-day the dentists the world over would also become instrumental in creating a work which would constitute the universal history of dental science. Dr. Förberg submitted this proposition to the consideration of the Executive Council. It was decided that this question should be settled at the next meeting in Madrid.

The Council then adopted the by-laws of the Federation, with the exception of a slight modification affecting the financial side and resulting from the report presented by Drs. Aguilar and Sauvez with reference to the payment of fees.

The Council also decided to introduce a modification in the laws of the Federation regarding the regular and adjunct members of the Council, in order to make it possible to appoint the delegate from Russia a regular member of the Executive Council.

The secretary-general proposed that the Council should organize several international commissions,—one on schools, one on jurisprudence, and one on statistics. The Council then discussed the question of forming an international commission on jurisprudence by the appointment of one member in each country, and that the officers of the Executive Council should prepare a report of this question to be submitted at the next meeting in Madrid.

It was then decided that the discussions should be continued in Madrid, and that the officers of the Federation should have in charge the organization of the forthcoming meeting of the International Dental Federation, to be held in Madrid in April, 1903.

The Executive Council then adjourned.

(To be continued.)

